# Association of living arrangements with depressive symptoms among older adults in China: a cross-sectional study

**DOI:** 10.1186/s12889-019-7350-8

**Published:** 2019-07-29

**Authors:** Yong Zhang, Zifeng Liu, Lingling Zhang, Paiyi Zhu, Xin Wang, Yixiang Huang

**Affiliations:** 1 Department of Health Policy & Management, School of Public Health, Sun Yat-sen University, 74 Zhongshan 2nd Road, Guangzhou, Guangdong 510080 People’s Republic of China; 2 Department of Pathology, the 3rd Affiliated Hospital, Sun Yat University, 600 Tianhe Road, Guangzhou, Guangdong China; 3 Department of Nursing, College of Nursing and Health Sciences, University of Massachusetts, Boston, MA USA; 4 Health Development Research Center, School of Public Health, Sun Yat-sen University, 74 Zhongshan 2nd Road, Guangzhou, Guangdong China

**Keywords:** Depressive symptoms, Living arrangements, Older adults, Elderly, CHARLS, China

## Abstract

**Background:**

Depression is regarded as a major public health concern in our society. While living arrangements as a structural factor of social support may contribute to older adults’ depression. Our study aims to investigate the association between living arrangements and depressive symptoms among older adults in the whole China, and to explore whether such influences differ by genders.

**Methods:**

Data were obtained from the 2015 China Health and Retirement Longitudinal Study. The sample was comprised of 6001 individuals aged ≥60 years. Depressive symptoms were measured by the 10-item Short-Form Center for Epidemiological Studies Depression. Independent variables were divided into 4 groups, considering living with/without a spouse and living with/without a child. The multivariate logistic regression was used to estimate the relationship between living arrangements and depressive symptoms in four models.

**Results:**

Compared with living only with a spouse, people living with a spouse and child, or living alone were more likely to have depressive symptoms (odds ratio = 1.23 95% CI 1.06–1.42 and 1.40 95% CI 1.03–1.92, respectively). Women were more associated with depressive symptoms (odds ratio = 2.13), but there were no significant associations between living arrangements and depressive symptoms among women. Men living with a spouse and a child had stronger positively depressive symptoms (odds ratio = 1.37).

**Conclusions:**

Older adults living alone, or living with both a child and spouse were more likely to have depressive symptoms. It is important to provide more social services for those older adult, particularly for men living with a spouse and child.

**Electronic supplementary material:**

The online version of this article (10.1186/s12889-019-7350-8) contains supplementary material, which is available to authorized users.

## Background

Depression is regarded as a major public health concern and may become the second most common cause of disability by 2020, trailing only heart disease [1]. According to the World Health Organization (WHO) report, nearly 350 million people were affected by depression worldwide [2]. Depression causes great suffering, decreases physical and social functioning, and even increases the risk for suicide among the elderly [3, 4]. As the population is rapidly aging in China, it appeals to aware the importance of depression as an public health issue across the nation. According to the China Health and Retirement Longitudinal Study (CHARLS), nearly 40% of older adults aged 60 and over have reported depressive symptoms. [4]

Depression, the major chronic disease currently, has been proved to be associated with genetic [5], behavioral, physical activity [6], quality of sleep [7] and the health condition like chronic diseases [5]. In addition, evidence suggests that social support is an important contributor to depression [3]. Living arrangements as a structural factor of social support may contribute to older adults’ depression.

The association between depression and living arrangements had been studied previously all over the world. And these results may differed across societies and cultures. It’s noteworthy that the studies in western countries focused more on the living arrangements whether living alone or not had different effect on depressive symptoms. An American study showed that older people living alone had more depressive symptoms than those living with others [8]. Another study in Finland also found the same evidence that persons living alone and living with others were more likely to have depressive disorder than people living with spouse [9]. However, there are more different structure of living arrangements in Asian. Most previous studies have suggested that elderly living with child are associated with higher risk of depression overseas: in Singapore, people living alone and living with children are associated with higher depressive symptom scores [10]. In Korea, older adults living alone, living with an unmarried child, living with grandchildren are more likely to have depressive symptoms [11]. In Thailand, having child living in the district predicted a higher odds of depression in the elderly [12]. Another study has found an inverse relationship that living with child is a protective factor to the prevalence of depressive symptoms [13].

However, the evidence for the relationship between living arrangements and depressive symptoms in China were very limited. And in Silverstein et al. study, it had drawn conflicting conclusions: older adults living with three-generation or living with grandchild were less likely to have depressive symptoms [14].

Based on traditional Chinese culture and the existence of only one child, parental depression might be more serious in China [14]. Since the implementation of the universal two-child policy put forward by the Chinese government in 2015 [15], parents bear a heavier burden to care for their children. Therefore, we researched whether older adults living with children may be associated with negative psychological states.

Previous studies have shown the association between depression and gender, marital status, physical health status in older adults [5, 16–18]. However, the association between the living arrangements of older adults and depression is conflict in different studies.

What’s more, it has been recognized in many studies that depressive symptoms vary by gender among different living arrangements. In Taiwan, women living alone were more detrimental to be depressive symptoms [19]. In Vietnam, older male living with child had more positive effect in psychological wellbeing than female [20]. While in Singapore, male living alone with weak social networks had higher depressive symptom scores [10]. However, little was known in China about the association between living arrangements and depressive symptoms by different gender. Many studies in China just identified that female seemed to have higher risk of depressive symptoms than male [21–23], and we cannot find the gender effect of association between living arrangements and depressive symptoms.

Therefore, our study aimed to investigate the association between living arrangements (including whether living with a spouse and whether cohabitating with a child) and depressive symptoms by gender, exploring whether older adults living with children may have more depressive symptoms, verifying whether the influence of such symptoms differs by gender, and considering what our society should do to improve mental health in older adults.

## Methods

### Study population

Data were derived from the China Health and Retirement Longitudinal Study (CHARLS), a nationally representative cohort study, including a mix of urban and rural settings and a wide variety of levels of economic development, followed up every 2 years since 2011 to serve the needs of scientific research on the elderly aged 45 and older The samples were selected through multistage probability sampling. Firstly, it was chosen by PPS, a probability-proportional-to-size sampling technique from a sampling frame containing all county-level units within 28 provinces with the exception of Tibet. These samples were stratified by eight regions, by whether they lived in urban district or rural county, and by county-level GDP [8]. Secondly, we chose 3 primary sampling units (PSUs) from each county-level unit (villages in rural areas and urban communities in urban areas) by PPS [24]. Thirdly, we selected dwellings within each PSU at random. Finally, it includes 28 provinces, 150 counties, and 450 communities [25, 26].

**Fig. 1:**
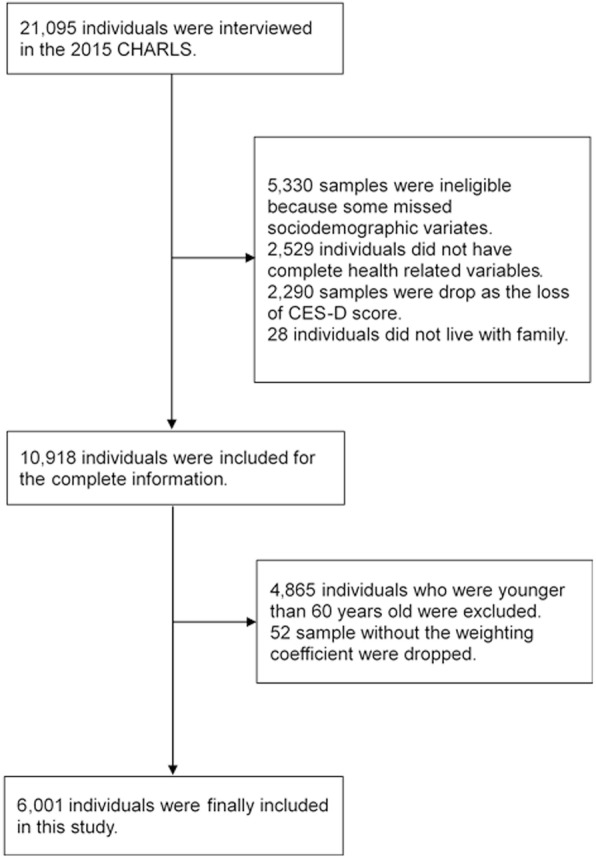
Participants’ flow in the study

We used the 2015 CHARLS involved 21,095 respondents as a cross-sectional study excluding 5,330 individuals who did not have completed sociodemographic data, 2529 individuals who missed health related information so the sample for our study was reduced to 13,236. In our current analyses, 2,290 sample missed CES-D score and 28 individuals who did not live with family were drop. A total of 4865 individuals who were younger than 60 years old were excluded as we focused on older adults like other studies [27]. Finally 6,001 individuals were included in our study, excluding 52 samples without weighting coefficient (Fig. 1). The individuals included in our study had to have at least one living child and live with family to exclude other influences of depression on older people who had no children.

Our current study is a secondary analysis of publicly available data from the CHARLS survey, which can be downloaded from the following Web site: http://charls.pku.edu.cn/zh-CN/page/data/2015-charls-wave4 It was approved by the Ethics Committee of Peking University Health Science Center [28].

### Study variables

#### Outcome variable: depressive symptoms

Depressive symptoms were measured with the 10-item Short-Form Center for Epidemiological Studies Depression (CES-D) [29]. This scale has been used in many studies with high reliability and validity [30, 31], especially in Chinese elderly. The score of this scale ranged from 0 to 30 with 10 items on a four-point Likert scale [0 = rarely or none of the time (< 1 day); 1 = some or a little of the time (1–2 days); 2 = occasionally or a moderate amount of the time (3–4 days); 3 = most or all of the time (5–7 days)]. There were two reverse-scoring negatively keyed items in this scale. In order to have better cross-study comparability, we used the cut-off score of 10 or above which had been used in previous studied using CHARLS data [24, 32] and shown an expected positive correlation with poorer health status scores [33] to identify the presence of significant depressive symptoms. Thus, depressive symptoms were divided into a binary variable (0 = does not have depressive symptoms; 1 = has depressive symptoms). In our study, the Cronbach’s alpha for these 10 items was 0.76 [16].

#### Main independent variable: living arrangements

Since the cohabitation status of both spouses and children was important in our study, we examined living arrangements by the interaction between cohabiting with spouses and children. Therefore, our main independent variables were: (1) living with a spouse but not a child, (2) living with a spouse and a child; (3) not living with a spouse or a child; and (4) not living with a spouse but living with a child.

#### Covariates

The covariates included sociodemographic variables, health behaviors, and health conditions. For sociodemographic factors, gender was dichotomized as female and male. Ages were grouped into 60–69, 70–79, and 80 years and over [21]. Marriage was defined as married and unmarried (including people who widowed, divorced or never married.) Education level was assessed in four groups: illiterate, primary school, middle school, and high school or above. Area was dichotomized as rural and urban.

Health behaviors were measured by smoking, drinking, and social activity [34]. We defined 0 = *non-smoker, non-drinker*, 1 = *ex-smoker*, *sometimes alcohol user*, and 2 = *current smoker*, *often alcohol user*. We considered social activity as dichotomous. 0 = *no socially activity* and 1 = *participant who was involved in at least one kind of socially activity*. In the definition of social activity, it included 12 kinds of activities asked in the questionnaire whether the respondent had done in the last month.

For health conditions, BMI, ADL disability, self-reported health, and the number of chronic diseases were used. BMI (body mass index) were used to define general obesity [32]. We divided respondents into 4 groups according to WHO definition [35]: underweight (< 18.5 kg/m2), normal (18.5 to 24.9 kg/m2), overweight (25 to 29.9 kg/m2), and obese (≥30 kg/m2). ADL disability (lack of ability to perform activities of daily living) was described as difficulty associated with six items: dressing, bathing, eating, getting into or out of bed, using the toilet, and continence control. We defined it as dichotomous as ADL-disabled if they could not complete one of these items by themselves. Self-reported health was first evaluated by participants in five levels (*very good, good, fair, poor, very poor*). We integrated *‘good’* for *‘very good’*, *‘good’*, or *‘fair’* and *‘poor’* for *‘poor’* or *‘very poor’*. We assessed the number of chronic diseases by asking each participant to select from a list of 14 chronic diseases (hypertension, dyslipidemia, diabetes, cancer, chronic lung diseases, liver disease, heart problems, stroke, kidney disease, digestive disease, psychiatric problems, memory-related disease, arthritis or rheumatism, and asthma). We divided this variable into 4 groups: 0 = *no chronic diseases*, 1 = *one chronic disease*, 2 = *two chronic diseases*, and 3 = *more than two chronic diseases*.

### Statistical analysis

We calculated the weighting coefficient in all variables, based on the non-response-adjusted weight to reduce the non-response bias. Descriptive statistics were used to calculate the number and proportion of the distribution by whether they have depressive symptoms or not. All variables were detected by χ2 to analyze the difference in the distribution between the case group (have depressive symptoms) and the control group (not have depressive symptoms).

We used multivariate logistic regression to estimate the odds ratio of the prevalence of depressive symptoms in different living arrangements adjusting for other covariates, first separately or in sets, and finally for all variables simultaneously. After that, we tested the association between living arrangements and depressive symptoms stratified by gender.

In sensitivity analysis, considering the definition of depressive symptoms was different in different studies, we repeated the analysis of logistic regression using another classification of depressive symptoms which the cut-off point in CESD was 16 [36]. The result confirmed the consistent association between living arrangements and depressive symptoms (Additional file 1: Table S1). In addition, after we controlled sociodemographic variables in Model 2, we added the health behavior variables in Model 3. And the health conditions were entered in the logistic regression model (Model 4) to find the association between living arrangements and depressive symptoms. All odds ratios (OR) were recorded with the 95% confidence interval (CI). The analyses were performed with STATA 14.0.

## Result

Table 1 presents the descriptive characteristics of our sample. Of the total number of participants, 3,050 were men (57.02%) and 2,951 were women (42.98%). Nearly 35.94% were measured as having depressive symptoms (28.13% for men and 44.02% for women). 41.29% lived with a spouse but not a child, while 35.14% lived with both a spouse and a child. Only 12.36 and 11.20% lived without a spouse with/without a child, respectively. Sociodemographic variables revealed that a large majority (66.89%) was 60–69 years old. Nearly 80% had low education levels, and more than four-fifths of participants lived in rural areas. Without any adjustment, we found that living arrangements was associated with depressive symptoms (*p* < 0.05). The covariates (sociodemographic variables, health behavior and health condition) included in the baseline were all associated with depressive symptoms respectively (*p* < 0.05) expect for the age group.

**Table 1:** Distribution of study variables overall

Variable		Total *N* = 6001		Depressive Symptoms^b^ *N* = 2157		No Depressive Symptoms *N* = 3844		*p*-value^a^
		n	%	n	%	n	%	
Living arrangements								
Living with spouse	living with child							0.000
Yes	no	2,478	41.29	790	36.62	1,688	43.91	
	yes	2,109	35.14	756	35.05	1,353	35.20	
No	no	672	11.20	287	13.31	385	10.02	
	yes	742	12.36	324	15.02	418	10.87	
Sociodemographic variables								
Gender								0.000
	male	3,050	57.02	858	39.78	2,192	57.02	
	female	2,951	42.98	1,299	60.22	1,652	42.98	
Age								0.158
	60–69	4,014	66.89	1,453	67.36	2,561	66.62	
	70–79	1,661	27.68	603	27.96	1,058	27.52	
	80-	326	5.43	101	4.68	225	5.85	
Marriage								0.000
	married	4,876	81.25	1,662	77.05	3,214	83.61	
	unmarried/widowed/divorced	1,125	18.75	495	22.95	630	16.39	
Education								0.000
	Illiterate	1,861	31.01	823	38.15	1,038	27.00	
	Primary school	2,843	47.38	1,040	48.22	1,803	46.90	
	Middle school	860	14.33	212	9.83	648	16.86	
	High school or above	437	7.28	82	3.80	355	9.24	
Area								0.000
	rural	4,796	79.92	1,868	86.60	2,928	76.17	
	urban	1,205	20.08	289	13.40	916	23.83	
Health behavior								
Smoking								0.000
	no	3,283	54.71	1,291	59.85	1,992	51.82	
	quit	776	12.93	239	11.08	537	13.97	
	now	1,942	32.36	627	29.07	1,315	34.21	
Drinking								0.000
	no	1,594	26.56	464	21.51	1,130	29.40	
	seldom	443	7.38	157	7.28	286	7.44	
	often	3,964	66.06	1,536	71.21	2,428	63.16	
Phsical activity								0.000
	no	2,953	49.21	1,160	53.78	1,793	46.64	
	yes	3,048	50.79	997	46.22	2,051	53.36	
Health condition								
BMI^c^								0.000
	underweight	472	7.87	218	10.11	254	6.61	
	normal	3,673	61.21	1,334	61.85	2,339	60.85	
	overweight	1,571	26.18	506	23.46	1,065	27.71	
	obse	285	4.75	99	4.59	186	4.84	
ADL disability^d^								0.000
	independent	5,569	92.80	1,886	87.44	3,683	95.81	
	dependent	432	7.20	271	12.56	161	4.19	
Self-reported health								0.000
	good	1,675	27.91	284	13.17	1,391	36.19	
	poor	4,326	72.09	1,873	86.83	2,453	63.81	
Chronic diseases								0.000
	0	1,424	23.73	363	16.83	1,061	27.60	
	1	1,662	27.70	549	25.45	1,113	28.95	
	2	1,340	22.33	495	22.95	845	21.98	
	≥3	1,575	26.25	750	34.77	825	21.46	

^a^The chi-square test was used for calculating the *P* value, to evaluates the association between depressive symptoms and exposure factors, depressive symptoms and covariates

^b^Depressive symptom, based on CES-D10 scores of 10-item Short-Form Center for Epidemiological Studies Depression

^c^BMI, body mass index

^d^ADL disability, activities of daily living disable

Table 2 shows the results of the multivariate logistic regression analysis between living arrangements and depressive symptoms. We found that different living arrangements had statistic difference in depressive symptoms. Crude OR’s shows that living without either spouse or child, and living without spouse but with child had higher risk of depressive symptoms than only living with spouse (OR 1.61, 95% CI 1.32–1.96 / OR 1.59, 95% CI 1.30–1.94). After controlling for the sociodemographic variables in model 2, we found that only older adults living with a spouse and a child had positive significantly effect in depressive symptoms, compared with those living only with a spouse (OR 1.15, 95% CI 1.00–1.33). However, model 3 showed that there was no difference between living arrangements and depressive symptoms adjusting for both sociodemographic variables and health behavior (including smoking, drinking and social activity). When controlling for all confounding variables (containing sociodemographic, health behavior and health conditions), the association between people living with both spouse and child, living without neither spouse nor child and depressive symptoms improved in magnitude. Model 4 showed that older adults living with only a spouse had the lowest risk of having depression tested by the multivariate logistic regression analysis. Compared with living with a spouse only, people living without neither a spouse nor a child and living with both a spouse and a child were more likely to have depressive symptoms (OR 1.40, 95% CI 1.03–1.92; OR 1.23, 95% CI 1.06–1.42). Even though individuals living only a child were 33% more likely to have depressive symptoms than people living with only a spouse, the proportion was nearly not statistically significant. In addition, the following variables were associated with stronger depressive symptoms: being female, younger age group, lower education levels, living in rural areas, current smoker, little social activity, lower BMI, having ADL disability, worse self-related health, and more chronic diseases shown in Table 3.

**Table 2:** Logistic regression analysis of the relationship between living arrangements and depressive symptoms in 4 Models

Variable		Model 1^a^		Model 2^b^		Model 3^c^		Model 4^d^	
		OR	95% CI	OR	95% CI	OR	95% CI	OR	95% CI
Living arrangements									
Living with Spouse	Living with child								
Yes	no	1.00		1.00		1.00		1.00	
	yes	1.20**	(1.05,1.39)	1.15*	(1.00,1.33)	1.15	(1.00,1.32)	1.23**	(1.06,1.42)
No	no	1.61***	(1.32,1.96)	1.30	(0.98,1.74)	1.29	(0.96,1.73)	1.40*	(1.03,1.92)
	yes	1.59***	(1.30,1.94)	1.20	(0.87,1.65)	1.19	(0.86,1.66)	1.33	(0.94,1.88)

^a^Model 1 was univariate binary logistic regression analysis of relationship of living arrangements and depressive symptoms

^b^Model 2 was based on Model 1 adjusting for the sociodemographic variables: gender, age, marriage, education level and region

^c^Model 3 was based on Model 2 adjusting for the health behavior: smoking, drinking and social activity

^d^Model 4 was based on Model 3 adjusting for the health conditions: BMI, ADL disability, self-reported health and chronic diseases

*p < 0.05, **p < 0.01, ***p < 0.001

**Table 3:** Logistic regression analysis of the relationship between living arrangements and depressive symptoms adjusted by all covariates

Variable		OR	95% CI
Living arrangements			
Living with spouse	Living with child		
Yes	No	1	
	Yes	1.23**	(1.06,1.42)
No	No	1.40*	(1.03,1.92)
	Yes	1.33	(0.94,1.88)
Sociodemographic variables			
Gender			
Male		1	
Female		2.13***	(1.75,2.60)
Age			
60–69		1	
70–79		0.91	(0.77,1.07)
≥ 80		0.56***	(0.41,0.77)
Marriage			
Married		1	
Unmarried/widowed/divorced		1.11	(0.80,1.54)
Education level			
Illiterate		1	
Primary school		0.89	(0.77,1.04)
Middle school		0.65***	(0.52,0.82)
High school or above		0.54***	(0.39,0.76)
Area			
Rural		1	
Urban		0.63***	(0.51,0.78)
Health behavior			
Smoking			
No		1	
Quit		1.15	(0.90,1.46)
Now		1.28**	(1.06,1.56)
Drinking			
No		1	
Seldom		1.18	(0.89,1.57)
Often		1.03	(0.85,1.25)
Social activity^a^			
No		1	
Yes		0.84**	(0.73,0.95)
Health condition			
BMI			
Underweight		1	
Normal		0.80*	(0.64,1.00)
Overweight		0.58***	(0.45,0.75)
Obse		0.54**	(0.37,0.78)
ADL disability^b^			
Independent		1	
Dependent		2.65***	(2.08,3.39)
Self-reported health			
Good		1	
Poor		3.15***	(2.68,3.70)
Chronic disease			
0		1	
1		1.25*	(1.04,1.50)
2		1.34**	(1.10,1.62)
≥ 3		2.06***	(1.71,2.49)

^a^Reference categories for social activity is people who had never done any social activities in the last month

^b^Reference categories for ADL disability is people who could finish activities of daily living without any difficulties

*p < 0.05, **p < 0.01, ***p < 0.001

Considering the prevalence of depressive symptoms was 2.13 times higher than male, Table 4 reveals a gender-stratified analysis regarding the association between living arrangements and depression. Adjusted for all covariates, depressive symptoms were strongest in men living with a spouse and a child (OR 1.37, 95% CI 1.12–1.68) compared with those living with a spouse only. But there was no significant association between living arrangements and depressive symptoms for women.

**Table 4:** ^a^ Adjusted effect of living arrangements on the depressive symptoms by sex

Variable	Living arrangements			
Gender	Living with spouse		Not living with spouse	
	Not living with child	Living with child	Not living with child	Living with child
Male^b^				
OR	1.00	1.37**	1.35	1.31
95%CI		(1.12,1.68)	(0.84,2.16)	(0.77,2.25)
Female^b^				
OR	1.00	1.12	1.50	1.45
95%CI		(0.91,1.38)	(1.00,2.26)	(0.94,2.24)

^a^Adjusted for sociodemographic (gender, age, marriage, education level and region), health behavior (smoking, drinking, social activity) and health condition (BMI, ADL disability, self-reported health and chronic diseases)

^b^Logistic regression group by sex

^*^p < 0.05, ^**^p < 0.01, ^***^p < 0.001

## Discussion

In this study, we aimed to investigate the association between depressive symptoms and living arrangements in Chinese older adults and to distinguish the difference between men and women. Our results showed that elderly people living with a spouse and a child, or living without a spouse nor a child had higher odds for depressive symptoms compared with people living with only a spouse. We also confirmed women were more likely to have depressive symptoms but they had no significant association between living arrangements and depressive symptoms. Men living with a spouse and child were more likely to have depressive symptoms.

The results of the present study demonstrated that only living with spouse was least likely to have depressive symptoms, which is consistent with results of earlier studies [9, 36, 37]. As Chappell et al. claimed having a spouse is “greatest guarantee of support in old age” in 1991, having children in households does not add too much health benefits [38].

We examined living with/without a spouse and living with/without a child as independent variables. We found a direct relationship between such living arrangements and depressive symptoms. This association was most apparent in older adults who lived without a spouse nor a child or lived without a spouse but with a child. Since older adults were special group depending more on social support [10], living without a spouse nor a child (means living alone), was more likely to lead to depression because there was minimum social interaction. Owing to lower levels of social support for those living without spouses [39], they cannot share their emotions with another person, which may lead to mental problems. Although the odds ratio of people living alone decreased from 1.61 to 1.40 after adjusting all covariate, such living arrangement still had the highest risks of having depressive symptoms.

In addition, living with a spouse and a child had statistically significant on the risk of depressive symptoms in our study. This is a contradictory phenomenon compared to previous studies. Previous studies had reported that older adults living with a child [14, 40] were less likely to have depression. Probably, cohabitating with children may give older people a sense of pride for Chinese culture, as well as instrumental and emotional support [38]. However, in our study, the age and participants were inconsistent with previous studies which made the result contrary. During our study, the elderly might feel burdened by childcare and housework rather than receiving support from the adult child, and more conflicts might arise between and among family members in a multi-generational household [11]. It can be hypothesized that a parent who lives with a child and a spouse has to pay more attention to caring for both children and spouse, and performs housework.

During the logistic regression analysis in our study, we found that the association between living arrangements and depressive symptoms was statistically significant after controlling several covariates. We also discovered that the association still existed when we changed the cut-off point in Additional file 1: Table S1 We could draw a conclusion that such models were sensitive and robust because the association in our study was valid although we added different variables in the model and changed the coding in dependent variable.

However, the association become meaningless when we controlled the health behavior in Model 3. Taking such result into consideration, we found that the primary association in our study didn’t change in the four models, meaning that living arrangements had positive effect on depressive symptoms even though the OR became insignificant in Model 3. What’s more, the 95% CI of living with a spouse and child was (1.00, 1.32) which was so close to 1.00, meaning that such influence may be significant once we expand our sample size.

The results of our study were concordant with those of a previous study showing that women are more likely to have depressive symptoms [11, 36]. But there was no statistical significant between living arrangements and depressive symptoms in women. In terms of regular gender roles, based on traditional Chinese values, women are more likely to be responsible for the family, so they might be more likely to have depression [10, 11, 36]. However, there was no significant association between living arrangements and depressive symptoms for women. Women in China have always felt a greater responsibility to care for their families, no matter who lived with them. Therefore, the composition of the domestic unit had little impact on them. Nevertheless, men living with a spouse and a child had the strongest depressive symptoms. This may be because older men in China have always occupied the main position in the family and society. Therefore, when they live with a child and spouse, they would overthink their child, their spouse and their family’s circumstances, which might make them anxious and depressed.

Similar to previous studies, we confirmed that low SES (Socioeconomic status) especially lower education levels were more likely to have depressive symptoms [41]. And the health conditions including ADL disability, self-rate health, and the number of chronic diseases were strongly associated with depressive symptoms, similar to previous reports [10, 18, 22, 41]. To make our model more preferably, we adjusted the BMI as a covariate health condition factor in our study which was fewer included in the model. The odds of people living with a spouse and child increased from 1.15 to 1.23 after adjusting the health conditions. Although it was not our focus, we can draw a conclusion that health conditions played an important role in depressive symptoms especially in the elderly living with a spouse and child.

We identified several political implications in our study. As older people seems to be more vulnerable to loneliness and social isolation, we should pay more attention to their mental health [42] especially for people living alone or living with a spouse and child. Regarding the community, which had been proved to be the most accessible way to expand the social coverage in the older nowadays [43], the infrastructure and healthcare facilities in community should be kept more attention to be improved by policymakers because such aspects had been prove to be more likely to lead to depression in Chinese older adults [44]. In addition, since men living with a spouse and a child were more likely to be depressed, different social services should be provided according to different kinds of household composition. We should not only focus on the mental health in older people, but also concern with the child and their spouse who would influence their psychological state directly.

The strength of our study is the use of a national sample among older adults in China, so our conclusions are more representative. Previous studies had focused on either rural or urban China [14, 18, 21], but our study focused on the whole areas, making our conclusions more universal.

Several limitations of our study should be noted, however. First, the data we used in this survey were cross-sectional, not necessarily causal. We could not draw conclusions about whether depressive symptoms were due to living arrangements. In addition, we can’t avoid the occurrence of endogeneity which may come from the genetic, reverse causality and so on. However, we have made the endeavor to decrease the effect of the potential variates by adding the covariates, which had been proved to have effect on depression previously, as much as possible in Model 4. We had tried to avoid the endogeneity by sensitivity analysis we done above. Second, as our study was secondary data, the influence factor in our model is circumscribed. We focused on the association between living arrangements and depressive symptoms. So many factor likes the mainly subjective loneliness which had been proved as a predicted factors increased the depression scores were not taken into account [45]. Thus further study containing more potential risk factors should be explored in the future.

## Conclusions

Older adults living alone or living with a spouse and child are at higher risk to have depressive symptoms. Men living with a spouse and a child are more likely to have depressive symptoms. Finding from our discovery in China may have implications for other countries which have undergone rapid urbanization and aging that older adults living with child is the main family support system. Government should better complete the age-old system of providing social services, particularly for the infrastructure and healthcare facilities in community. Different housed composition should be provided different social services not only for the older but also for their cohabitant.

## Aditional file

Additional file 1 : **Table S1** Logistic regression analysis of the relationship between living arrangements and depressive symptoms adjusted by all covariates. (DOCX 15 kb)

## Data Availability

The data in this study is publicly available from Peking University at a free charge. It can be downloaded from the following Web site: http://charls.pku.edu.cn/zh-CN/page/data/2015-charls-wave4 accurately.

## Abbreviations

ADL disability: Activities of daily living disable
CES-D10: Scores of 10-item Short-Form Center for Epidemiological Studies Depression
CHARLS: China Health and Retirement Longitudinal Study
GDP: Per capita statistics on gross domestic product
PPS: A probability-proportional-to-size sampling technique
PSUs: Primary sampling units
WHO: World Health Organization
