# Supplementary material for: Association of living arrangements with depressive symptoms among older adults in China: a cross-sectional study
Source: BMC Public Health. 2019 Jul 29;19:1017. doi: 10.1186/s12889-019-7350-8 (PMC6664592; doi:10.1186/s12889-019-7350-8)
Supplement: Supplementary file 1 — : Table S1 Logistic regression analysis of the relationship between living arrangements and depressive symptoms adjusted by all covariates. (DOCX 15 kb) [file 12889_2019_7350_MOESM1_ESM.docx]

| **Supplemental Table 1** Logistic regression analysis of the relationship between living arrangements and depressive symptoms adjusted by all covariates. | | | |
| --- | --- | --- | --- |
| **Variable** | | **OR** | **95% CI** |
| **Living arrangements** | |  |  |
| **living with spouse** | **living with child** |  |  |
| yes | no | 1.00 |  |
|  | yes | 1.21* | （1.00,1.46） |
| no | no | 1.54 | （1.07,2.24） |
|  | yes | 1.33 | （0.89,1.98） |
| **Sociodemographic variables** | |  |  |
| **gender** | |  |  |
| male | | 1.00 |  |
| female | | 1.84*** | （1.44,2.35） |
| **age** | |  |  |
| 60-69 | | 1.00 |  |
| 70-79 | | 0.91 | （0.75,1.10） |
| ≥80 | | 0.49** | （0.32,0.75） |
| **marriage** | |  |  |
| married | | 1.00 |  |
| unmarried/widowed/divorced | | 1.28 | （0.88,1.85） |
| **education level** | |  |  |
| Illiterate | | 1.00 |  |
| Primary school | | 0.81* | （0.67,0.97） |
| Middle school | | 0.48*** | （0.34,0.66） |
| High school or above | | 0.38*** | （0.22,0.65） |
| **area** | |  |  |
| rural | | 1.00 |  |
| urban | | 0.64** | （0.49,0.83） |
| **Health behavior** | |  |  |
| **smoking** | |  |  |
| no | | 1.00 |  |
| quit | | 1.09 | （0.80,1.47） |
| now | | 1.17 | （0.91,1.49） |
| **drinking** | |  |  |
| no | | 1.00 |  |
| seldom | | 1.72** | （1.22,2.42） |
| often | | 1.32* | （1.05,1.67） |
| **social activity^a^** | |  |  |
| no | | 1.00 |  |
| yes | | 0.80** | （0.67,0.94） |
| **Health condition** | |  |  |
| **BMI** | |  |  |
| underweight | | 1.00 |  |
| normal | | 0.72* | （0.55,0.95） |
| overweight | | 0.53*** | （0.39,0.72） |
| obse | | 0.36*** | （0.23,0.58） |
| **ADL disability^b^** | |  |  |
| independent | | 1.00 |  |
| dependent | | 3.35*** | （2.59,4.33） |
| **self-reported health** | |  |  |
| good | | 1.00 |  |
| poor | | 3.38*** | （2.65,4.30） |
| **chronic disease** | |  |  |
| 0 | | 1.00 |  |
| 1 | | 1.19 | （0.92,1.53） |
| 2 | | 1.28 | （0.99,1.69） |
| ≥3 | | 2.21*** | （1.72,2.84） |
| ^a^Reference categories for social activity is people who had never done any social activities in the last month. | | | |
| ^b^Reference categories for ADL disability is people who could finish activities of daily living without any difficulties. | | | |
| *p<0.05, **p<0.01, ***p<0.001 | | | |
